# Evaluate Typhoon Disasters in 21st Century Maritime Silk Road by Super-Efficiency DEA

**DOI:** 10.3390/ijerph16091614

**Published:** 2019-05-08

**Authors:** Xiaobing Yu, Hong Chen, Chenliang Li

**Affiliations:** 1 Collaborative Innovation Center on Forecast and Evaluation of Meteorological Disasters, Nanjing University of Information Science & Technology, Nanjing 210044, China; 2 School of Management Science and Engineering, Nanjing University of Information Science & Technology, Nanjing 210044, China; 20141347048@nuist.edu.cn (H.C.); 20151307047@nuist.edu.cn (C.L.); 3 Silicon Lake College, Kunshan 215332, China

**Keywords:** typhoon disaster, vulnerability, super-efficiency DEA model, The Belt and Road Initiative

## Abstract

The Belt and Road Initiative involves many countries and areas. As the introducer, China plays a key role in the initiative. However, the coastal areas in China have frequently been hit by typhoons that lead to huge casualties and economic losses. In order to reduce damages caused by natural disasters, this paper selected the coastal regions of the 21st Century Maritime Silk Road as the study areas, specifically Shanghai, Zhejiang, Guangdong, Fujian, and Hainan, to estimate the vulnerability to typhoon disasters based on the historical data about typhoon disasters and the super-efficiency data envelopment analysis (DEA) evaluation model. Although Shanghai is a low-vulnerable region, it needs to pay close attention to the risk of typhoon disasters due to the outstanding economic influence. In addition, it was found that the vulnerability to typhoons in Zhejiang, Guangdong, and Hainan showed a dramatic fluctuation from 2011 to 2016, and Zhejiang’s vulnerability in 2013 was extremely high compared to other years. Meanwhile, Guangdong and Hainan are highly vulnerable areas, suffering from typhoon disasters heavily. Moreover, the vulnerability to typhoons for Fujian is relatively low.

## 1. Introduction

A Silk Road Economic Belt and a 21st Century Maritime Silk Road were proposed by Chinese President Xi Jinping in September and October 2013, respectively [1]. At present, it is referred to as the Belt and Road Initiative, which integrates the historical symbolism of the ancient Silk Road with the new requirements of today. The Belt and Road Initiative features mutual respect and benefit and aims to promote international economic governance toward a fair, just, and rational system, which connects over 65 countries in Asia, Europe, and Africa. The total economic volume of the related countries exceeds 21 trillion US dollars [2]. The proposal of the Belt and Road is inspiring and may lead to a win–win situation with tremendous economic benefits to various countries if implemented smoothly [3]. The 21st Century Maritime Silk Road is the new extension of Silk Road, committed to the joint development and utilization of marine resources with countries along the route, and projects to strengthen the circulation of elements, such as commodity, trade, capital, and culture. In collaboration with other countries, especially developing countries, the initiative of 21st Century Maritime Silk Road is beneficial to creating new regional business relationship. At the same time, it is also conducive to alleviating the situation of overcapacity in China and promoting industrial upgrading and transformation. However, the natural environment through the Maritime Silk Road is relatively harsh. Natural disasters like typhoons, floods and thunderstorms hit countries along the line frequently, and the economic losses caused by natural disasters to these areas are enormous. Carrying out research on natural disasters is of great significance for the government’s disaster prevention and mitigation, which can reduce disaster damage and social impact. For instance, China’s coastal areas have suffered lots of typhoon disasters that have resulted in considerable casualties and property damages [4]. Particularly, China’s coastal area of the 21st Century Maritime Silk Road, namely Shanghai, Zhejiang, Guangdong, Fujian, as well as Hainan, is the most advanced area and is highly vulnerable to typhoons. In order to minimize the risk of typhoon disasters for coastal regions, it is necessary to focus on research work about aspects of natural hazards [5,6,7], risk management [8,9,10], related health issues [11,12] and the vulnerability of each region to natural disasters [13,14].

A standardized and comprehensive hazard vulnerability assessment can help healthcare facilities to identify and stratify potential hazards [15]. Vulnerability assessment methods in the context of climate change and natural disasters were discussed by Birkmann et al., and two case studies were used to contrast the opportunities and current constraints in scenario methods at different scales [16]. With the support of GIS spatial functions, Ma et al. tested farmers’ vulnerability to floods in the Poyang Lake Region [17]. Until now, a lot of studies on vulnerability to disaster have mainly focused on the frameworks about vulnerability [18,19,20,21,22] and varied vulnerability to climate change [23,24,25,26,27,28]. For instance, the vulnerability of India and Indian states to climate change was assessed using the Vulnerability–Resilience Indicator Prototype [29]. Silva et al. carried out an investigation with respect to the relationship between floods, droughts, and the socioeconomic groups at a local level in Sri Lanka. As a result, the low-income households that make a living through natural resources are more likely to suffer from flood and drought disasters and undergo economic losses [30]. Moreover, Ye et al. applied entropy weight theory into typhoon disaster vulnerability assessment and plotted the spatial distribution map of typhoon disaster vulnerability for the Fujian province of China [31].

China’s Belt and Road initiative belongs to the world. Currently, many scholars are discussing the initiative from different perspectives. The Belt and Road initiative of China provides a great opportunity for promoting the Resourcing Future Generations program across much of the Eurasian continent [32]. Under the background of the Belt and Road Initiative, a measurement equation for China’s exports to the five Central Asian countries based on the gravity model of international trade is performed to forecast China’s future export growth potential [33]. Zhang et al. found the regional carbon emissions intensity in Belt and Road initiative nations increases in 2013–2015, and thermal power is a priority to develop [34]. Motivation, implications, and other countries’ response to the Belt and Road Initiative have been analyzed [35,36,37,38].

In a word, the new Silk Road initiatives create a medium of communication for companies and capital to make a series of investments in other countries by taking advantage of China’s strengths in infrastructure, financial power, and manufacturing capacity [39]. Yet, there have been few studies about the natural disasters related to the countries along the Belt and Road, especially for typhoon disasters. Furthermore, as the Belt and Road Initiative is implemented, studies on the natural disaster are also supposed to be abundant. Meanwhile, China, as the initiator of the initiative, itself is plagued by natural disasters. The coastal region suffers a lot from natural disasters, especially typhoon disasters [40,41]. Therefore, keeping in view of the research gap, we took the major coastal regions of the 21st Century Maritime Silk Road as the case study to evaluate the vulnerability to typhoon disasters using the super efficiency data envelopment analysis. It is a novel perspective that enriches the work on natural disasters along the Belt and Road. The findings can further help the local government to put forward a specific policy recommendation to improve the ability against typhoon disasters. Further, it can provide great reference value and practical significance for disaster prediction, as well as enhance the rescue capability in meteorological disasters.

## 2. Materials and Methods

At present, various methods are widely used in research, such as field investigation, the historical recording method, the indicator framework, and the method of analogue function. Among them, field investigation uses questionnaires and face-to-face interviews to conduct sample surveys on the affected individuals that mainly focus on vulnerable individuals with high vulnerability, neglecting the total vulnerability of the affected population and reducing the reliability of the overall measurement. The indicator framework is to establish an evaluation system in advance and then select different kinds of indicators, determine the weights of them through the Delphi method, gray correlation method, analytic hierarchy process, etc., which finally conducts a comprehensive assessment of vulnerability. Unfortunately, due to the lack of consensus about the selection principle and empowerment of indicators, it may lead to the absence of credibility. In addition, the method of analogue function is applied into the risk assessment for accurately and quantitatively assessing disaster vulnerability. However, it needs to assume a functional model in advance and takes a large amount of data as the support. The results are easily affected by the presupposed model.

**Figure 1:**
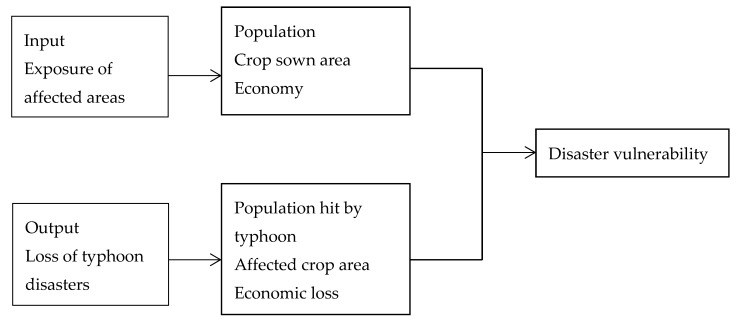
Input–output analysis of vulnerability to typhoon disasters.

Based on historical data about disaster, the historical recording method is applicable to assess vulnerability at the macro scale. The disaster risk index (DRI) and HOTSPOTS program are representative applications of this method. Specifically, the DRI program uses the ratio of the number of people killed by natural disasters to exposures as an index of relative vulnerability [42]. Meanwhile, the HOTSPOTS program takes the population mortality and economic loss ratio to evaluate disaster vulnerability [43]. Referring to the idea, this paper takes the vulnerability to typhoon disasters in China’s major coastal regions of the 21st Century Maritime Silk Road as the objective. Influenced by both natural and economic systems, the single ratio cannot represent disaster vulnerability. In our paper, we consider typhoon disaster vulnerability as the result of multiple inputs and outputs. On the basis of the historical recording method, we took the exposure of affected areas as the input and the loss of typhoon disasters is regarded as output. Finally, vulnerability to typhoon disasters is measured by the ratio of input to output. Further detail is shown as follows in Figure 1.

Data envelopment analysis (DEA) is an efficiency evaluation method proposed by American operation researchers [44]. It can be used to measure the relative efficiency of multi-input and multi-output for decision-making units. DEA does not need to set parameters in advance and only needs to evaluate the decision-making unit according to the input–output data, avoiding the intervention of subjective factors, using explicit indicators to give evaluation to the decision-making unit. The traditional DEA evaluation model is shown as follows, where $x_{jm}$ represents the value of mth input indicator of jth region, and $y_{jn}$ represents the value of nth output indicator of jth region. Further,$\;s_{m}^{-}$ and $s_{n}^{+\;}\;$are the slack variables of input surplus and output deficiency, respectively, *ε* is Archimedes infinitesimal, and$\;w_{j}$ represents the weight of indicator. 1/$\theta_{k}$ is the relative efficiency value of the kth decision unit.

(1) $$\max\;\left[\theta_{k}-\varepsilon (\overset{t}{\underset{m=1}{\sum }}s^{-}+\overset{r}{\underset{n=1}{\sum }}s^{+})\right]=v_{d}\left(\varepsilon \right)$$

(2) $$s.t.\{\begin{array}{c} \overset{n}{\underset{j=1}{\sum }}x_{jm}w_{j}+s_{m}^{-}=x_{km}\\ \overset{n}{\underset{j=1}{\sum }}y_{jn}w_{j}-s_{n}^{+}=\theta_{k}y_{kn}\\ w_{j}\geq 0;s_{m}^{-}\geq 0;s_{n}^{+}\geq 0\\ j=1,2,\cdots n;\;\\ n=1,2,\cdots r;\\ m=1,2,\cdots t \end{array}$$

The traditional DEA model fails to distinguish the decision-making units with the efficiency value equals to the highest value “1”. To solve the problem, some researchers have proposed the super-efficiency DEA model [45]. In order to effectively assess the vulnerability to typhoons of selected regions, this paper adopts the super-efficiency DEA model, which is shown as follows:

The $x_{jm}$ represents the value of mth exposure indicator of jth region, while $y_{jn}$ represents the value of nth loss indicator of jth region. Meanwhile,$\;s_{m}^{-}$ and $s_{n}^{+}\;$are the slack variables and *ε* is Archimedes infinitesimal, while $w_{j}$ represents the weight of indicator. 1/$\theta_{k}$ is the relative efficiency value of the kth decision unit. The improvement of the super-efficient DEA model is replacing the data of ith decision-making unit with the linear combination of other decision-making units when evaluating the efficiency of it. In this paper, the higher the efficiency value is, the greater the vulnerability of the decision-making unit to typhoon disaster.

(3) $$\max\;\left[\theta_{k}-\varepsilon (\overset{t}{\underset{m=1}{\sum }}s^{-}+\overset{r}{\underset{n=1}{\sum }}s^{+})\right]=v_{d}\left(\varepsilon \right)$$

(4) $$s.t.\{\begin{array}{c} \overset{n}{\underset{j=1,j\neq k}{\sum }}x_{jm}w_{j}+s_{m}^{-}=x_{km}\\ \overset{n}{\underset{j=1,j\neq k}{\sum }}y_{jn}w_{j}-s_{n}^{+}=\theta_{k}y_{kn}\\ w_{j}\geq 0;s_{m}^{-}\geq 0;s_{n}^{+}\geq 0\\ j=1,2,\cdots n;\;\\ n=1,2,\cdots r;\\ m=1,2,\cdots t \end{array}$$

To estimate the vulnerability to typhoon disasters, the coastal regions of the 21st Century Maritime Silk Road as the case study were selected as the study areas, namely Shanghai, Zhejiang, Guangdong, Fujian, and Hainan. Based on the input–output system, considering the availability of data and referring to [46,47], this paper chose the representative indices to vulnerability assessment. The detailed information is as follows in Table 1.

The data were collected from the China Statistical Yearbook, China Meteorological Yearbook (2012–2017), annual climate bulletins of various regions, and information provided by departments. Particularly, due to the lack of a record about Shanghai’s typhoon disaster loss in 2014, the mean of the two years before and after was adopted to replace the missing one.

## 3. Results

**Figure 2:**
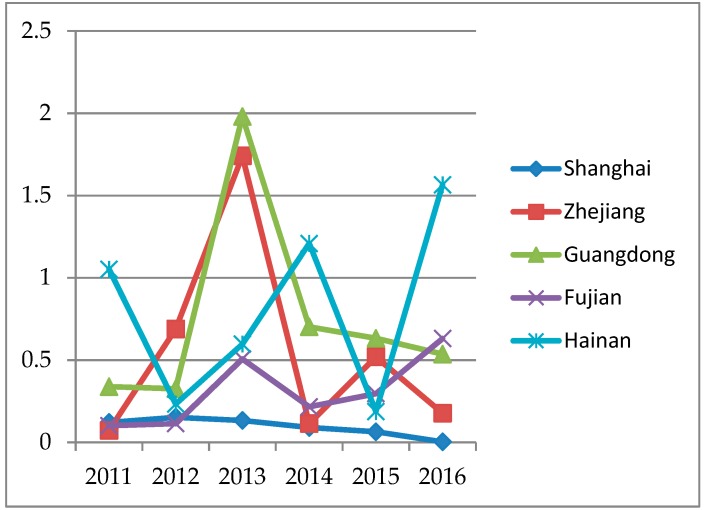
The change of vulnerability to typhoon.

The vulnerability to typhoon disasters of selected areas is shown in Table 2. The higher the relative efficiency value is, the greater the vulnerability of region to typhoon. That means the comprehensive loss caused by a typhoon to the area is more serious compared to others in the same situation. Further, Table 2 indicates that the vulnerability to typhoons of Zhejiang, Guangdong, and Hainan is significantly higher than that of Shanghai and Fujian on the whole. Among them, the typhoon vulnerability of Zhejiang is extremely higher than in previous years due to the huge impact of super typhoons like Haikui in 2012, Fitow in 2013, and Soudelor and Chan-hom in 2015. Nine typhoons landed on or affected Guangdong in 2013, especially Usagi and Utor; such kinds of super typhoons caused a lot of damage to Guangdong. As shown in Figure 2, the changes in the vulnerability of typhoon disasters in Shanghai and Fujian from 2011 to 2016 are relatively stable. As the area that is frequently hit by typhoon in China, Hainan has been in a highly vulnerable state in the past six years.

## 4. Discussion

In order to further analyze the vulnerability to typhoon disasters of selected areas, this paper compared the exposure, loss, and vulnerability in each region in the past six years in Table 3. Shanghai ranked third and fourth in the typhoon disaster vulnerability in 2011 and 2012, respectively, and its typhoon disaster vulnerability has been at the end since then. In general, Shanghai is a high exposure–low loss–low vulnerability area, but it is still necessary to strengthen the typhoon disaster warning and prevention work. As the center of economy and culture in the Yangtze River Delta, Shanghai ranks top in the country, with a large number of people and enterprises gathering here, and it will lead to huge losses as soon as a typhoon disaster occurs. Although only a few typhoons have landed in Shanghai over the years, the direct economic losses caused by this have been extremely serious. Therefore, Shanghai should effectively improve the risk assessment of natural disaster and emergency rescue work to reduce the loss of a catastrophe disaster chain in metropolitan areas. 

Overall, Zhejiang was in a state of high exposure–medium loss–medium vulnerability from 2011 to 2016. As a coastal, economically developed region, Zhejiang is always in the forefront of five regions in terms of population, economy, and crop sown area. Thus, Zhejiang is a high-exposure area. In the past six years, Zhejiang has ranked among the top three in the vulnerability of typhoon disasters three times. Moreover, Guangdong belongs to the high exposure–high loss–high vulnerability area, and its typhoon disaster vulnerability is always at the top. Among the five research objects, the direct economic losses caused by typhoon disasters in Guangdong, the largest economic aggregation, have remained stable in the top two five times over the years. It can be seen that typhoons have a particularly destructive effect on the Guangdong’s economy. As the core of the Pearl River Delta, it ought to think highly of the system of typhoon disasters, improve the collaboration capability of meteorological, oceanic, transportation, and civil affairs departments to deal with major natural disasters, and build an effective disaster emergency response and rescue system.

In addition, the vulnerability of typhoon disasters in Fujian is low generally, and it has low exposure–low loss–low vulnerability. Due to the super typhoons Nepartak, Meranti, and Megi landing in Fujian in 2016, major casualties and property losses caused the rank of vulnerability to rise up to the second spot. Meanwhile, Hainan had the highest vulnerability to typhoon disasters in 2011, 2014, and 2016, ranking first among five regions. Hainan is a region with low exposure–high loss–high vulnerability on the whole. In the face of a typhoon of the same intensity, Hainan may suffer from more serious disaster losses due to its high vulnerability. 

Table 4 reflects the ranking of vulnerability to typhoon disasters and the selected regions were divided into three categories. The first echelon of vulnerability was Guangdong and Hainan, which represents high disaster vulnerability. Zhejiang is the only one in the second echelon with medium class. Meanwhile, there were two regions in the third tier, namely Shanghai and Fujian, which reflect low typhoon disaster vulnerability. With a large population density and per capita GDP, Guangdong is more vulnerable to typhoon disasters. That means that when faced with a typhoon of the same level, Guangdong may suffer more losses. Further, it has the maximum crop sown area among the selected objects. Once hit by typhoon, this can lead to the vast damage to farmers. The government needs to make overall land plans for agriculture and increase farmers’ awareness of disaster risk. Hainan is always subject to typhoons in summer, which results in the huge loss of population and crop. Considering the high vulnerability of Hainan, the improvement of prediction about typhoon disasters is essential in order to reduce the damage. Generally speaking, the vulnerability of Zhejiang has declined over time because of the excellent emergency plan for typhoon defense. In 2012, the Zhejiang Meteorological Bureau issued the “Operational Regulations for Typhoon Report” for the first time that officially standardized services about typhoons, resulting in great progress in governance of predisaster warning. Further, the vulnerability to typhoons of Shanghai remained stable from 2011 to 2016, which represented a state of low vulnerability. Yet, the risk management of disaster cannot be ignored by the government on account of the remarkable advantage in economy. Moreover, Fujian’s vulnerability is relatively low among the chosen regions. With the exception of 2016, Fujian’s vulnerability retained a low level. As the region is often hit by typhoons, public meteorological services should be further provided in various forms to maximize the coverage of information about disasters in order to keep the public informed.

## 5. Conclusions

Based on the historical recording method, this paper took the coastal areas of the 21st Century Maritime Silk Road in China as the research object and used the super-efficiency DEA model to evaluate the vulnerability to typhoon disasters in Shanghai, Zhejiang, Guangdong, Fujian, and Hainan between 2011 and 2016. Furthermore, the exposure of these regions, loss caused by typhoons, and overall vulnerability were analyzed in detail. The results indicated that the vulnerability to typhoons in Zhejiang, Guangdong, and Hainan showed a dramatic fluctuation from 2011 to 2016. Although Shanghai is a low-vulnerable city, it needs to pay close attention to the risk of typhoon disasters, enhance the ability to respond to natural disasters, and prevent sudden-onset disasters because of its large population and production activities. In addition, with it having been hit by typhoons such as Soulik, Kong-Rey, and Fitow, the vulnerability of Zhejiang Province in 2013 was extremely high compared with other years. Meanwhile, Guangdong and Hainan are highly vulnerable areas, and typhoon disasters have caused huge casualties and economic losses. The vulnerability to typhoons for Fujian is relatively low. Hainan is often attacked by typhoons in China, so the rank of vulnerability has always been in the forefront over the past years, especially in 2011, 2014, and 2016.

Typhoon disasters have become the main obstacle to the sustainable development of the economy in coastal areas of China. The formulation of disaster prevention and reduction measures is an important guarantee for sustainability. Based on the analysis of our study, suggestions are given in the following part.

The successful implementation of prediction about typhoon disasters is inseparable from advanced technology and equipment. Studies about high-accuracy typhoon monitoring instruments are supposed to be improved and enhance the exactitude of meteorological disaster prediction. At the same time, the refined management of disaster prediction systems is carried out, and the responsibilities of each post should be standardized clearly so as to achieve a comprehensive regulatory system. In addition, the establishment of an information sharing platform ought to be pushed on in order to increase the efficiency of typhoon disaster prediction.

Improving the emergency response mechanism of typhoon disasters is conducive to reducing losses caused by natural disasters and enhancing the rescue capability of meteorological disasters. It is necessary to strengthen the uniformity of emergency command and rescue work and ensure related information transfer quickly among different departments. In particular, information disclosure should be given great importance, which means the public should have easy access to it. Meanwhile, closely monitoring the condition of typhoons in metropolitan areas and comprehensively assessing the risk of disasters are beneficial to the construction of an emergency support system.

The propaganda campaign needs to be enriched and make the public aware of the fatalness of catastrophic weather such as typhoons and heavy rain in order to take proper measures. Uniting available social forces and making full use of resources such as the government, media, and research institutes will comprehensively strengthen the public’s awareness of typhoon so that they can exert themselves to play a part in the process of rescue.

## Figures and Tables

**Table 1:** Selection of index.

Factor	Variable of Input	Variable of Output
Population	Population density	Affected population
Agriculture	Crop sown area	Affected crop area
Economy	Per capita GDP	Economic loss

**Table 2:** The vulnerability to typhoon disasters of selected areas from 2011 to 2016.

Area	Vulnerability					
	2011	2012	2013	2014	2015	2016
Shanghai	0.121	0.152	0.133	0.09	0.064	0.003
Zhejiang	0.072	0.688	1.741	0.114	0.519	0.177
Guangdong	0.338	0.326	1.98	0.702	0.632	0.536
Fujian	0.101	0.114	0.507	0.216	0.296	0.631
Hainan	1.052	0.233	0.597	1.209	0.187	1.565

**Table 3:** The exposure and loss.

		Exposure			Loss		
Time	Area	Population Density/People Per Square Kilometer	Per Capita GDP/Ten Thousand Yuan Per Person	Crop Sown Area/Hectare * 1000	Affected Population/Ten Thousand	Economic Loss/Billion Yuan	Affected Crop Area/Hectare * 1000
2011	Shanghai	3701.89	8.32	400.60	34.60	2.50	7.00
	Zhejiang	517.82	5.92	2462.70	97.80	23.10	15.00
	Guangdong	584.59	5.07	4572.00	261.20	36.60	287.00
	Fujian	300.00	4.72	2285.80	105.20	10.60	50.40
	Hainan	247.74	2.88	838.30	553.53	68.92	223.50
2012	Shanghai	3753.94	8.64	387.90	42.00	5.20	14.70
	Zhejiang	519.15	6.34	2324.20	891.20	275.50	378.00
	Guangdong	589.54	5.39	4629.60	310.00	44.30	295.20
	Fujian	302.26	5.26	2263.10	93.43	15.60	59.41
	Hainan	250.56	3.22	854.60	131.05	13.09	62.90
2013	Shanghai	3809.15	9.22	377.30	12.10	3.70	28.00
	Zhejiang	521.14	6.87	2311.90	1234.70	609.00	613.00
	Guangdong	592.32	5.87	4698.10	2147.70	421.80	984.00
	Fujian	304.35	5.79	2292.20	313.20	103.60	258.15
	Hainan	252.83	3.55	848.20	352.90	30.40	156.00
2014	Shanghai	3826.50	9.92	357.00	13.65	3.00	18.00
	Zhejiang	522.09	7.29	2274.00	158.50	10.80	57.00
	Guangdong	596.77	6.32	4744.90	554.50	255.30	700.00
	Fujian	306.94	6.32	2305.20	70.90	16.50	117.71
	Hainan	255.08	3.88	859.60	612.33	177.40	306.35
2015	Shanghai	3809.15	10.62	340.20	15.20	2.30	8.00
	Zhejiang	525.02	7.74	2290.50	667.60	219.50	368.00
	Guangdong	603.73	6.71	4784.70	613.50	288.00	617.00
	Fujian	309.60	6.77	2331.30	268.50	88.60	120.83
	Hainan	257.34	4.06	845.30	112.60	12.40	36.00
2016	Shanghai	3817.03	11.64	294.70	0.46	0.24	0.31
	Zhejiang	529.86	8.45	2274.40	231.23	92.07	98.07
	Guangdong	612.08	7.23	4830.80	295.67	343.90	60.68
	Fujian	312.42	7.36	2327.30	310.94	179.13	286.09
	Hainan	259.04	4.42	823.30	457.90	76.70	459.30

**Table 4:** The rank of vulnerability to typhoon disasters.

	Shanghai	Zhejiang	Guangdong	Fujian	Hainan
2011	3	5	2	4	1
2012	4	1	2	5	3
2013	5	2	1	4	3
2014	5	4	2	3	1
2015	5	2	1	3	4
2016	5	4	3	2	1
